# Distinct Associations of Motor Domains in Relatives of Schizophrenia Patients—Different Pathways to Motor Abnormalities in Schizophrenia?

**DOI:** 10.3389/fpsyt.2018.00129

**Published:** 2018-04-23

**Authors:** Lea Schäppi, Katharina Stegmayer, Petra V. Viher, Sebastian Walther

**Affiliations:** University Hospital of Psychiatry, University of Bern, Bern, Switzerland

**Keywords:** neurological soft signs, fine motor function, relatives of schizophrenia, motor function, dimension, schizophrenia

## Abstract

**Introduction:**

Aberrant motor function is an integral part of schizophrenia. In fact, abnormalities are frequently found in patients, in populations at risk, and in unaffected relatives. Motor abnormalities are suspected to be relevant for the clinical outcome and could probably predict the conversion from at-risk individuals to schizophrenia. Furthermore, motor function has been argued as endophenotype of the disorder. Yet, which particular motor domain may classify as a potential endophenotype is unknown. We aimed to compare schizophrenia patients, unaffected first-degree relatives and healthy controls for different motor domains. We expected impairments in all domains in patients and in some domains in relatives.

**Method:**

We included 43 schizophrenia patients, 34 unaffected first-degree relatives of schizophrenia patients, and 29 healthy control subjects, matched for age, gender, and education level. We compared motor function of four motor domains between the groups. The domains comprise neurological soft signs (NSS), abnormal involuntary movements (dyskinesia), Parkinsonism, and fine motor function including simple [finger tapping (FT)] and complex fine motor function, (i.e., dexterity as measured with the coin rotation test). Furthermore, we tested the association of motor function of the four domains with working memory, frontal lobe function, and nonverbal intelligence for each group separately using within-group bivariate correlations.

**Results:**

Schizophrenia patients showed poorer motor function in all tested domains compared to healthy controls. First-degree relatives had intermediate ratings with aberrant function in two motor domains. In detail, relatives had significantly more NSS and performed poorer in the FT task than controls. In contrast, complex fine motor function was intact in relatives. Relatives did not differ from controls in dyskinesia or Parkinsonism severity.

**Discussion:**

Taken together, schizophrenia patients have motor abnormalities in all tested domains. Thus, motor abnormalities are a key element of the disorder. Likewise, first-degree relatives presented motor deficits in two domains. A clear difference between relatives and healthy controls was found for NSS and FT. Thus, NSS and FT may be potential markers of vulnerability for schizophrenia. The lack of association between genetic risk and dyskinesia or Parkinsonism suggests distinct pathobiological mechanisms in the various motor abnormalities in schizophrenia.

## Introduction

Motor abnormalities constitute an integral part of schizophrenia. Aberrant motor function was included as one of eight dimensions of psychopathology in schizophrenia spectrum disorders in the current version of the diagnostic and statistical manual (DSM-5) (2014). Aberrant motor function was frequently observed in both medicated and unmedicated patients (1, 2). In addition, aberrant motor function often wax and wane during follow-up (3, 4). At least one motor sign was present in about 66% of patients in first-episodes (5), in 59% of patients on admission (6), and 80% of chronic patients (1, 7). Alterations incorporate various domains of motor function. Domains include for instance neurological soft signs (NSS) (i.e., motor coordination, sequencing, and sensory integration) (8), dyskinesia (abnormal involuntary movements typically manifest as involuntary continuous orofacial movements and dyskinetic movements of the extremities or trunk) (9, 10), parkinsonism (bradykinesia, rigor, tremor) (10), catatonic symptoms (pure motor signs, disturbance of volition, inability to suppress motor reactions and vegetative instability) [for review, see e.g., Ref. (1, 11, 12)], deficits in fine motor function [i.e., the coin rotation task, the Moberg pick-up test (13, 14), finger sequencing and the pegboard test (15)], and psychomotor slowing (16). In sum, schizophrenia patients show generalized aberrant motor function.

Another important line of evidence stems from first-degree relatives of psychosis patients. Aberrant motor function in these subjects may help find markers who are associated with the genetic risk for schizophrenia. However, a comprehensive assessment of a wide range of domains of motor function in first-degree relatives is missing. Thus, it remains unknown, which particular motor domain may serve as a potential genetic risk marker.

In detail, previous reports suggest delayed motor development as a genetic risk marker. For instance, it was hypothesized that NSS arise due to neurodevelopmental delay in motor function in young subjects at risk and first-degree relatives. NSS are subtle neurological deficits (17, 18) which are seen in children and normally vanish during the motor development in late childhood. An association of NSS with the genetic risk for schizophrenia has consistently been reported (19), possibly even predictive for the transition to psychosis (20). Nearly, all studies investigating NSS in unaffected first-degree relatives detected higher prevalence of NSS in relatives compared to healthy controls. This has been substantiated by recent meta-analyses (8, 21).

Whether dyskinesia and Parkinsonism are associated with the genetic risk to develop schizophrenia is less clear than for NSS (22, 23). Even though, most reports show increased incidence of dyskinesia and Parkinsonism in schizophrenia relatives, which has been confirmed in a recent meta-analysis (24). Yet, for instance, Tarbox and Pogue-Geile (25) detected no increased prevalence of dyskinesia in relatives. As for dyskinesia, from the available data, it is difficult to state whether Parkinsonism may serve as a genetic risk marker for psychosis. Some reports showed increased incidence in unaffected relatives (24, 26) with some inconsistency (27).

One further important domain of motor function is fine motor function (i.e., manual skills and dexterity) (28). Schizophrenia patients suffer from defective complex fine motor function, as measured by the Line Copying Task or the coin rotation test (14) as well as simple fine motor function as measured by the finger tapping (FT) task [e.g., Ref. (14, 29, 30)]. Fine motor impairments are already present at the beginning of the disorder in the first episode. These deficits seem to be relatively stable over time (31). Moreover, defective fine motor function may impact activities of daily living and may be associated with poor functional outcome as has been shown in other conditions (i.e., Parkinson’s disease) (32, 33) In general, a loss of complex fine motor skills may not solely be explained by elemental motor deficits such as motor slowing (bradykinesia) but might also reflect disturbances of more complex motor function such as selectively control and coordination of upper limb and specifically finger movements (34, 35). Whether fine motor function is linked to the genetic risk for psychosis is unclear. One single report detected aberrant simple fine motor function (measured with FT) in unaffected first-degree relatives of psychotic patients (36). Likewise another study found reduced complex fine motor function (i.e., tested with inserting long and short pins into holes in a platform) in subjects at risk (37). However, complex fine motor function of the fingers (i.e., finger dexterity measured with the coin rotation task) has not been tested.

In sum, there is considerable and consistent evidence for the association of NSS and the risk to develop schizophrenia. A moderate number of studies suggest an association of dyskinesia and Parkinsonism with schizophrenia risk with some inconsistency. In addition, only few studies addressed the association of complex fine motor function and psychosis risk. Moreover, former investigations focused only on one motor domain. Thus, which domain of motor function is associated with a genetic psychosis risk and how they are interlinked is still unknown.

Therefore, the aim of our study was to compare for the first time a comprehensive motor battery, including motor function in four domains (NSS, dyskinesia, Parkinsonism and fine motor function) in three well-characterized groups (patients, unaffected first-degree relatives and healthy controls). In line with the literature, we expected generalized aberrant motor function in schizophrenia patients. Further, we expected aberrant motor function in some but not all motor domains in unaffected first-degree relatives. In detail, we hypothesized increased incidence of NSS and aberrant fine motor function in unaffected relatives, but no increased incidence of dyskinesia and Parkinsonism.

## Materials and Methods

### Subjects

This study included 41 patients with schizophrenia, 34 first-degree relatives (parents, siblings, or children) and 29 healthy controls matched for gender, age, and education. All participants were right-handed as determined by the Edinburgh handedness inventory (38) and were native German speakers. This study was carried out in accordance with the recommendations of the local Ethics Committee (Kantonale Ethikkommission: KEK Bern) with written informed consent from all subjects.

Patients were in- and outpatients recruited at the University Hospital of Psychiatry, Bern and diagnosed according to DSM-5. Relatives were contacted via patients with schizophrenia and the Association of Schizophrenia Patients’ Relatives, Bern. Inclusion criterion for relatives was a history or presence of any physician-diagnosed schizophrenia spectrum disorder in at least one first-degree relative. Controls were volunteers recruited from the hospital staff and the community without first-degree relatives with schizophrenia spectrum disorders.

General exclusion criteria for all participants were substance abuse or dependence other than nicotine and past or current medical or neurological condition associated with impaired movement (i.e., dystonia, idiopathic Parkinsonism, or stroke).

All Participants were interviewed by trained psychiatrists using the Mini-International Neuropsychiatric Interview (39) (adapted for DSM-5). In addition, we assessed frontal lobe function using the Frontal Assessment Battery (FAB) (40), verbal working memory with the digit span backwards (DSB) task [subtest from the Wechsler Memory Scale (WMS-III)] (41) as well as nonverbal intelligence using the short Test of nonverbal Intelligence (TONI) (42).

### Motor Behavior

We assessed four different domains of motor function using a comprehensive battery including the neurological evaluation scale (NES) (18) to assess NSS, the Abnormal Involuntary Movement Scale (AIMS) (43) for dyskinesia, and the Unified Parkinson’s Disease Rating Scale (UPDRS) (44) for Parkinsonism. In addition, participants performed a complex and a simple fine-motor tasks. During the complex fine-motor task, the coin rotation task (CR), a coin (Swiss 50-Rappen coin, corresponding exactly in size to a US-Nickel) is rotated between thumb and fingers one and two (index and middle finger) in 10 s as fast as possible. A correction for drops is used [the adjusted score is the number of rotations in 10 s minus (0.1 × rotations × drops)] (34, 45, 46). After a short training period, each participant performed three trials of 10 s with each hand. Performance was videotaped and analyzed in slow motion by a blinded rater. The last half-turn was included when at least half of the movement was completed. Finally, participants performed a simple fine motor task, the FT task. They were instructed to tap their index finger and thumb with the maximum amplitude as fast as possible. This task was also performed for three times during 10 s with each hand. Again, performance was video recorded and analyzed by a blinded rater.

### Statistical Analysis

Statistical tests were performed using SPSS 24.0 (SPSS Inc., Chicago, IL, USA). Univariate analysis of variance, chi-square tests (χ^2^), and general linear models were used to test the continuous and categorical clinical variables between groups (relatives, patients and controls). Our main interest was to assess whether patients and relatives had more severe motor abnormalities than controls. Therefore, we first compared ratings of motor scales between groups using univariate analyses of variance. *Post hoc* tests included Sidak correction for multiple comparisons. Next, we calculated two separate repeated measures ANOVAs of performance in the two fine motor tasks with side (left and right hand) as within-subject factor. To test the association of aberrant motor function as well as working memory and frontal lobe function, we correlated scores of each scale and task for each group (patients, relatives and controls) separately, applying within-group bivariate correlations. Significance level was set at *p* < 0.05 two-tailed.

## Results

Clinical and demographic data are given in Table 1. Groups did not differ in age, gender, and education. However, groups differed regarding nonverbal intelligence and working memory, for which patients had inferior performance compared to relatives or controls. In addition, patients had poorer frontal lobe function at trend level (*p* = 0.081) compared to healthy controls (see Table 2).

**Table 1:** Demographic data and clinical status of the groups.

	Controls (C); *N* 29	Relatives (R); *N* 34	Patients (P); *N* 43	df	*F*/χ^2^	*p*-Value
Age	40.86 ± 14.38	42.74 ± 15.73	37.98 ± 11.37	2	1.18	0.312
Gender	55.2% (f)	64.7% (f)	62.8% (f)	2	0.67	0.768
EDU	13.79 ± 2.32	14.18 ± 3.02	13.52 ± 3.12	2	0.49	0.617
DOI (years)	–	–	12.92 ± 12.48			
PANSS tot	–	–	71.14 ± 16			
PANSS pos	–	–	17.98 ± 6.28			
PANSS neg	–	–	18.14 ± 5.15			

*N, number; f, female; EDU, education (years); DOI, duration of illness; PANSS, Positive and Negative Syndrome Scale; tot, total; pos, positive; neg, negative*.

**Table 2:** Nonverbal intelligence, frontal lobe and working memory function of the groups.

	Controls (C); *N* 29	Relatives (R); *N* 34	Patients (P); *N* 43	df	*F*/χ^2^	*p*-Value	*Post hoc* Sidak corrected
TONI	109.24 ± 10.50	108.15 ± 8.43	97.68 ± 11.40	2	14.28	**<0.001**	**P < C (***p*** < 0.001)** **P < R (***p*** < 0.001)**
FAB	17.17 ± 0.97	16.85 ± 1.54	16.10 ± 2.72	2	2.78	0.067	P < C (*p* < 0.081)
DSB	5.31 ± 0.71	5.26 ± 0.79	4.42 ± 1.05	2	9.45	**<0.001**	**P < C (***p*** = 0.001)** **P < R (***p*** = 0.001)**

*N, number; TONI, test of nonverbal intelligence (index scores); FAB, frontal assessment battery; DSB, digit span backwards*.

*Note: significant group differences are highlighted in bold*.

### Motor Function

**Figure 1:**
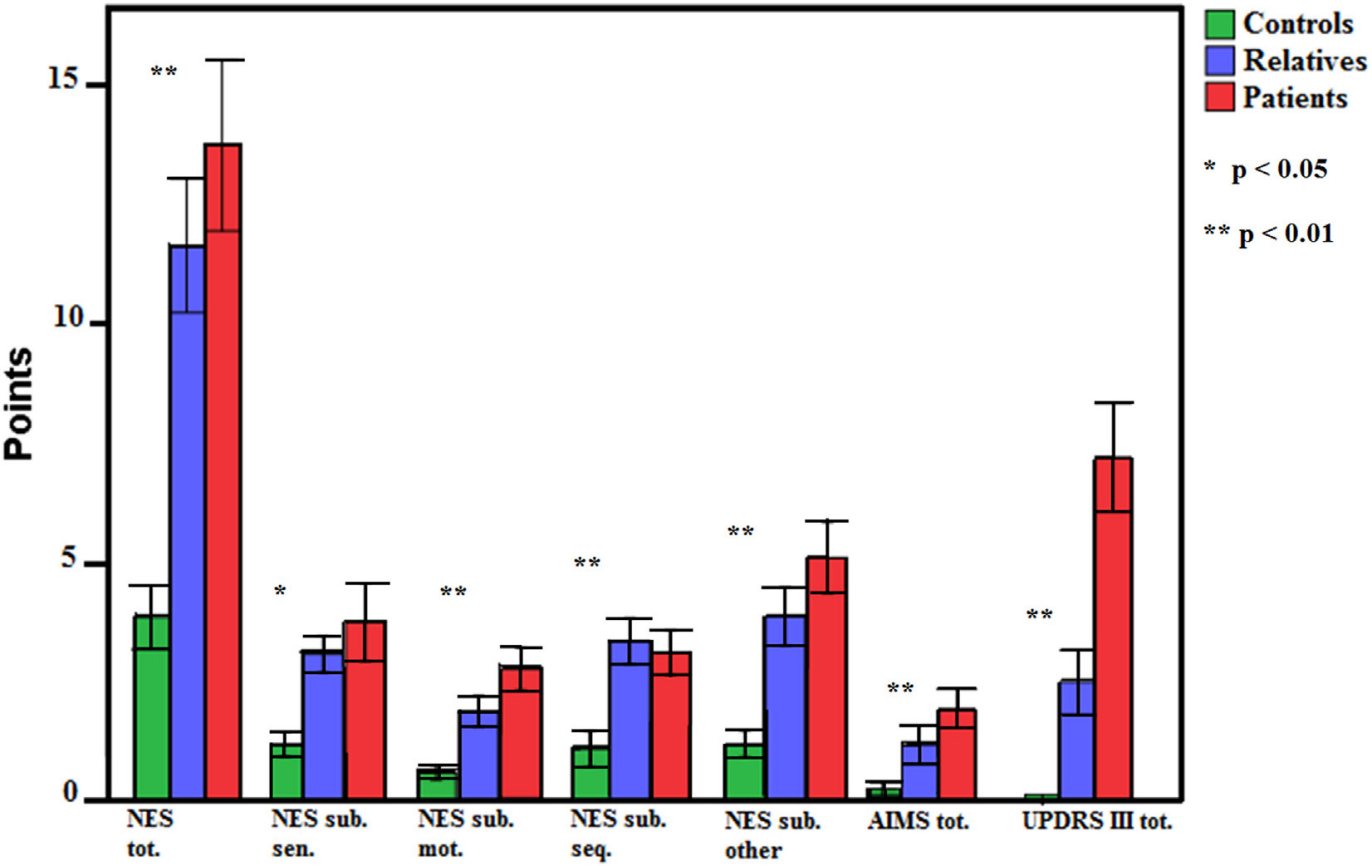
Group differences of neurological soft sign, dyskinesia, and Parkinsonism in patients, relatives, and controls. Error bars indicate SE. NES, Neurological Evaluation Scale; sub., subscale; sen., sensory motor function; mot., motor coordination; seq., motor sequencing; AIMS, Abnormal Involuntary Movement Scale; UPDRS III, Unified Parkinson’s Disease Rating Sale motor part III.

Groups differed in incidence of aberrant motor function in the applied motor scales (see Table 3 and Figure 1). In detail, patients and relatives had higher NSS ratings than healthy controls, indicating more pathological signs. Next, we explored group differences in NSS subdomains (NES subscales). Again, groups differed in all measured motor functions (sensory motor function, motor coordination, motor sequencing, and others). In detail, performance of patients was worse in all subscales compared to healthy controls. Relatives demonstrated inferior performance in “motor sequencing” and “others,” as well as a trend of inferiority in “sensory integration” (*p* = 0.067) compared to healthy controls. For dyskinesia and Parkinsonism differences became evident between patients and healthy controls, but not between relatives and controls.

**Table 3:** Group differences of neurological soft sign, dyskinesia, and Parkinsonism in patients, relatives, and controls.

	Controls (C); *N* 29	Relatives (R); *N* 34	Patients (P); *N* 43	*F*	*p*-Value	*Post hoc* Sidak corrected
NES total	3.90 ± 3.59	11.65 ± 8.18	13.74 ± 11.75	10.83	**<0.001**	**R > C (***p*** = 0.003)** **P > C (***p*** < 0.001)**
NES Sensory integration	1.14 ± 1.19	2.94 ± 2.15	3.58 ± 5.12	4.23	**0.017**	**P > C (***p*** = 0.015)**
NES Motor coordination	0.55 ± 0.78	1.79 ± 1.82	2.60 ± 2.84	8.09	**0.001**	**P > C (***p*** < 0.001)**
NES Sequencing	1.03 ± 1.96	3.21 ± 2.72	2.60 ± 2.96	6.39	**0.002**	**R > C (***p*** = 0.005)** **P > C (***p*** = 0.008)**
NES Others	1.14 ± 1.43	3.71 ± 3.44	4.88 ± 4.73	9.12	**<0.001**	**R > C (***p*** = 0.020)** **P > C (***p*** < 0.001)**
AIMS total	0.14 ± 0.52	1.15 ± 2.36	1.91 ± 2.7	5.62	**0.005**	**P > C (***p*** = 0.003)**
AIMS Facial expression	0.04 ± 0.19	0.27 ± 0.75	0.26 ± 0.74	1.3	0.277	
AIMS Lips and perioral area	0.10 ± 0.41	0.32 ± 0.73	0.23 ± 0.43	1.27	0.285	
AIMS Jaw	0.0 ± 0.0	0.09 ± 0.38	0.11 ± 0.40	1.03	0.363	
AIMS Tongue	0.0 ± 0.0	0.15 ± 0.58	0. 17 ± 0.51	1.31	0.276	
AIMS Upper extremity	0.0 ± 0.0	0.15 ± 0.50	0.37 ± 0.77	3.77	**0.027**	**P > C (***p*** = 0.024)**
AIMS Lower extremity	0.0 ± 0.0	0.0 ± 0.0	0.31 ± 0.68	6.79	**0.002**	**P > C (***p*** = 0.008)** **P > R (***p*** = 0.005)**
AIMS Neck/shoulders/hips	0.0 ± 0.0	0.12 ± 0.48	0.143 ± 0.43	1.23	0.296	
UPDRS III	0.10 ± 0.56	2.50 ± 3.93	7.21 ± 7.46	17.19	**<0.001**	**P > R (***p*** = 0.001)** **P > C (***p*** < 0.001)**

*AIMS, Abnormal Involuntary Movement Scale; NES, Neurological Evaluation Scale; UPDRS III, Unified Parkinson’s Disease Rating Sale motor part III*.

*Note: significant group differences are highlighted in bold*.

**Figure 2:**
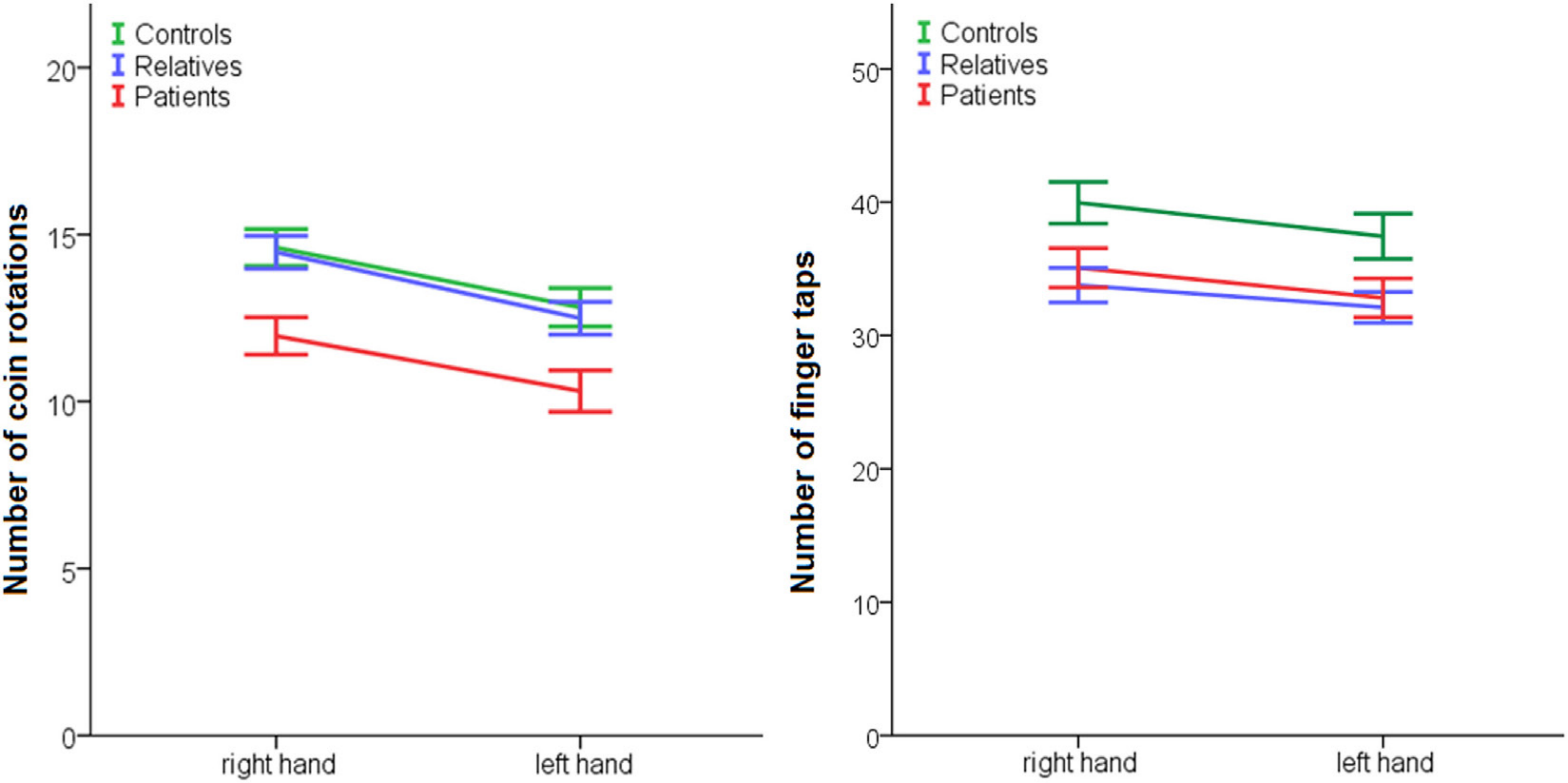
Performance of fine motor function in patients, relatives, and controls. Coin rotation: effect of group *p* = 0.001, effect of side *p* < 0.001, no effect side × group: *p* = 0.822; *post hoc*: patients < controls: *p* = 0.003; and relatives < controls *p* = 0.005. Finger tapping: effect of group: *p* = 0.018, effect of side: *p* < 0.001, no effect side × group: *p* = 0.646; post hoc: patients < controls: *p* = 0.061 and relatives < controls *p* = 0.022.

In addition, we tested complex (CR) and simple fine motor function (FT) (Table 4; Figure 2) exploring effects of side (left or right hand), group and group*side interactions. For CR, we found an effect of side in favor of the right hand, an effect of group but no group*side interaction. *Post hoc* Sidak tests indicated CR was impaired in patients compared to controls. Relatives did not differ from controls. Likewise, in FT, we detected an effect of side in favor of the right hand, an effect of group but no group*side interaction. *Post hoc* Sidak tests indicated that patients and relatives performed inferior to controls.

**Table 4:** Performance of fine motor function in patients, relatives and controls.

	Controls (C); *N* 29	Relatives (R); *N* 34	Patients (P); *N* 43
CR right (±SE)	14.61 ± 2.97	14.47 ± 2.87	11.96 ± 3.67
CR left (±SE)	12.82 ± 3.09	12.49 ± 2.87	10.31 ± 4.05
FT right (±SE)	39.95 ± 8.40	33.77 ± 7.55	35.07 ± 9.49
FT left (±SE)	37.44 ± 9.15	32.10 ± 6.75	32.82 ± 9.29

*CR, coin rotation; FT, finger tapping*.

### Association of Motor Function With Frontal Lobe Function and Working Memory

The association of motor domains including fine motor function with frontal lobe function and working memory is given in Table 5. Table 4 shows that ratings of some motor domains correlated with performance in motor tests. In fact, fine motor tests (CR and FT) are significantly associated with Parkinsonism in patients and unaffected relatives. Likewise, CR is significantly associated with NSS in patients. In contrast, dyskinesia (AIMS) seems to be independent of fine motor function (CR and FT). In addition, CR is unrelated to frontal lobe function in all groups and to working memory in relatives and controls. Furthermore, frontal lobe function is associated with NSS and Parkinsonism in all groups. Likewise, WM was significantly correlated with motor function in patients. This association is not seen in any other group.

**Table 5:** Association of motor function with frontal lobe function and working memory function in patients, relatives and controls.

	AIMS	UPDRS III	CR	FT	FAB	WM
**Patients**						
NES	0.357*	0.760**	-0.541**	−0.300	−0.628**	−0.479**
AIMS		0.303*	−0.266	−0.046	−0.122	−0.101
UPDRS III			−0.479**	−0.412**	−0.522**	−0.264
CR					0.195	0.349*
FT					0.077	0.116
FAB						0.492**
WM						
**Relatives**						
NES	0.283	0.356*	−0.403*	−0.438**	−0.399*	−0.299
AIMS		−0.060	−0.318	0.073	0.031	−0.378*
UPDRS III			−0.341*	−0.496**	−0.419*	−0.230
CR					0.144	0.072
FT					0.464**	0.017
FAB						0.307
WM						
**Controls**						
NES	0.471**	0.488**	−0.337	−0.271	−0.613**	−0.197
AIMS		0.694**	−0.041	−0.169	−0.623**	−0.121
UPDRS III			−0.104	−0.007	−0.432*	−0.084
CR					0.335	−0.155
FT					0.312	−0.027
FAB						0.127
WM						

*NES, Neurological Evaluation Scale; AIMS, Abnormal Involuntary Movement Scale; UPDRS III, Unified Parkinson’s Disease Rating Scale motor part III; CR, coin rotation; FT, finger tapping; FAB, frontal assessment battery; DSB, digit span backwards*.

*p < 0.05; **p < 0.01.

*Note: significant associations are highlighted in bold*.

## Discussion

Increasing evidence has established the clinical relevance of aberrant motor function in schizophrenia (1, 47–49). Here, we investigated a comprehensive motor battery with four domains of motor function in three well-characterized groups (patients, unaffected first-degree relatives and healthy controls). The study aimed to explore, which motor domains were particularly associated with the genetic risk for psychosis. We were able to confirm our main hypotheses, and demonstrated (1) generalized aberrant motor function in schizophrenia patients, and (2) aberrant motor function in some but not all motor domains in schizophrenia relatives. The distinct pattern of associations between genetic psychosis risk and motor domains argues for different pathophysiological mechanisms behind each motor domain.

As hypothesized, aberrant motor function was shown for NSS in schizophrenia relatives compared to controls with intermediate severity. Moreover, NSS are thought to indicate aberrant motor development that is even evident in adults. Aberrant motor development has been associated with a genetic psychosis risk (9). Thus, NSS have been discussed as endophenotype markers in psychosis (50). In contrast, we were not able to confirm our hypothesis of an association between genetic psychosis risk and defective coin rotation, which is a reliable and simple measure of complex fine motor function. In fact, in unaffected first-degree relatives fine motor function was preserved. However, in schizophrenia we detected aberrant complex fine motor function with the coin rotation task. In addition, patients and relatives performed worse than healthy controls in the simple fine motor task. Finally, relatives had similar severity of Parkinsonism and dyskinesia compared to healthy controls, which is different from the findings regarding NSS. Thus, our results confirm the specific importance of NSS for the genetic psychosis risk. Therefore, NSS may in the future be particularly useful to monitor vulnerable subjects.

### Generalized Aberrant Motor Function in Schizophrenia

Patients had an increased severity of NSS, dyskinesia and Parkinsonism. In addition, we detected aberrant fine motor function (FT and CR) in patients. Thus, in line with the literature we found generalized aberrant motor function in schizophrenia patients and extended the knowledge by demonstrating further alterations in complex fine motor function, which has been understudied. Complex fine motor function (i.e., manual dexterity as measured with the CR task) adopts an intermediate position between higher-order (i.e., apraxic) and elemental motor function. Aberrant complex fine motor function is characterized by impaired control of selective and coordinated hand and finger movements, not explained by weakness or sensory deficits (51). Aberrant function in this domain may impact performance of skilled movements in schizophrenia, such as hand gestures (14, 52–55). Similar deficits have previously been shown in Parkinson’s disease (35). Taken together, exploring the pathophysiology underlying complex fine motor function, incorporating instrumental approaches, may add to the innovative research in the field of motor function in schizophrenia.

### Aberrant Motor Function in Specific Motor Domains in Unaffected First-Degree Relatives

Unaffected first-degree relatives of schizophrenia patients showed aberrant motor function in some but not all investigated motor domains. In line with the literature, we found more NSS in relatives compared to healthy controls. Nearly all studies (56–60) and recent meta-analyses (8, 21) confirmed an increased severity of NSS in relatives of schizophrenia patients. Thus, NSS are very likely to be associated with the genetic risk for psychosis. However, some reports were negative, probably owing to inclusion of a considerable proportion of second-degree relatives (61) or the lack of specific NSS rating scales (62). Besides the total NSS severity, we detected differences between relatives and controls in two of the NES subscales, i.e., “sequencing of motor acts” (i.e., fist-ring test, the fist-edge-palm test, the Ozeretski test) and “others” (i.e., adventitious overflow, Romberg test, tremor, memory, mirror movements). Interestingly, a previous study reported increased heritability in the same subscales, with a correlation between NSS severity of patients and NSS severity of their siblings (56).

In contrast, relatives had no increased severity of dyskinesia and Parkinsonism in our sample. In line with this, some previous reports failed to detect increased dyskinesia in relatives (24, 25). Likewise, one study reported a relatively low incidence of parkinsonism (incidence 3% of the sample in 181 subjects) in unaffected relatives of schizophrenia patients (27). Yet, no control group was included in this latter study. In contrast, other reports (63) demonstrate an increased severity of dyskinesia (63) and Parkinsonism (26, 63) in schizophrenia relatives compared to healthy controls. Thus, whether dyskinesia or Parkinsonism is truly associated with the genetic risk for psychosis requires further clarification in larger samples. Alternatively, dyskinesia and Parkinsonism may exclusively occur in subjects at increased risk for psychosis, who have prodromal signs beyond a genetic risk (23, 64, 65).

Finally, while patients showed impaired complex fine motor function, in relatives, complex fine motor function (CR) was intact. No previous report investigated complex fine motor function (CR) in unaffected first-degree relatives. Currently, our findings argue for an association of impaired complex fine motor function with psychosis but not with genetic psychosis risk. In contrast to complex fine motor function and in line with one previous report, simple fine motor function was impaired in both patients and relatives and may, therefore, be associated with psychosis and the genetic risk (36).

To conclude, we demonstrated increased incidence of NSS and impaired simple fine motor function in schizophrenia relatives arguing for a particular relevance as a genetic risk marker. In contrast, dyskinesia, Parkinsonism and complex fine motor function were not impaired and are, therefore, unlikely to become relevant indicators of the genetic risk for psychosis.

### Aberrant Motor Function of Different Domains Linked to Distinct Alterations in the Motor System

In general, motor abnormalities in schizophrenia have been consistently associated with alterations in the cerebral motor system (48, 66–68). In addition, psychosis was associated with altered functional and structural connectivity in the motor system (68–77). Likewise, during motor tasks functional alterations in the motor system were detected in schizophrenia (78). Importantly, while there is some overlap, aberrant motor behavior of distinct motor domains is associated with specific structural and functional alterations (72, 79), i.e., not one mechanism associated with all domains of aberrant motor behavior in psychosis.

A large body of evidence associates NSS in schizophrenia with structural and functional alterations within cortical premotor and motor areas, the cerebellum and thalamus (48). In detail, NSS are linked to gray matter (GM) abnormalities in cortical areas comprising the pre- and postcentral gyri, premotor areas, inferior and middle frontal gyri (80, 81). Likewise, studies using cortical thickness or sulcation found NSS to be associated with aberrant morphology of frontal, temporal, and parietal areas (82, 83). Subcortical structures related to NSS in schizophrenia included the thalamus, caudate nucleus, putamen, globus pallidus (84, 85) as well as the cerebellum (80, 86, 87). Importantly, even medication-naive first-episode patients show an association of aberrant motor sequencing and GM alterations of cortical and subcortical structures (88). Interestingly, a longitudinal study further confirmed that persisting compared to decreasing NSS in first-episode schizophrenia is related to a progressive decrease of GM volume (89). White matter density has shown to be related to NSS in the inferior frontal gyrus, cerebellum, and corpus callosum in schizophrenia (80, 81). Furthermore, abnormal white matter cerebellar–thalamic tract development predicted NSS after 12 months in ultra-high risk individuals (90). Besides these structural abnormalities, some studies explored functional neural underpinnings of NSS (91). In a paradigm that compared the fist-edge-palm task with a simple motor task, patients with schizophrenia failed to activate the left middle frontal gyrus which was the case in healthy controls (92). In addition, with increasing task difficulty, functional connectivity changed between the sensorimotor cortex and the right frontal gyrus in patients compared to healthy controls (92). Thus, altered brain morphology and function may account for NSS in patients.

Brain imaging studies in schizophrenia and Parkinson’s disease reported associations of dyskinesia and Parkinsonism with basal ganglia dysfunction. For instance, patients with dyskinesia had reduced GM volume in the caudate nucleus, putamen, globus pallidus, and the thalamus (93). Likewise, altered volume of the striatum was shown in medication naïve chronic schizophrenia patients with dyskinesia (94) and volume alterations in the putamen were evident in subjects at risk with dyskinesia (95). Furthermore, white matter alterations within cortico-basal ganglia circuits correlated with involuntary movements in schizophrenia (96). Accordingly, early functional imaging studies reported that activity in the basal ganglia (higher relative metabolic rates in the putamen) was associated with dyskinesia severity in schizophrenia (97). In addition, D2/D3 receptor availability in the striatum was linked to Parkinsonism in schizophrenia (98). In addition, there is some conceptual overlap between Parkinsonism and reduced spontaneous motor activity, which is also paralleled by alterations of the cerebral motor system (72, 76, 77). The relatives in the current study had no increased severity of Parkinsonism. We may speculate that alterations in the suspected underlying brain areas are lacking in relatives. In contrast, basal ganglia shape abnormalities were also reported in unaffected siblings of schizophrenia patients (99). However, a recent study detected no alterations of caudate and putamen laterality indices in schizophrenia siblings, who did not significantly differ from controls (100) and abnormal striatal and ventricle volumes showed no sign of heritability (101). Thus, whether alterations in the basal ganglia are critical for dyskinesia and Parkinsonism in relatives of schizophrenia patients remains to be explored.

Finally, patients had significantly impaired complex fine motor function. Until now, no study investigated neural correlates of impaired coin rotation (complex fine motor function) in schizophrenia. In general, for complex fine motor function, temporal, and spatial information of the movement has to be retrieved and translated into motor output. According to modern models (102) brain areas of the so-called praxis network (103) are involved. Inferior parietal lobule (IPL) and intact connections between IPL and left premotor cortex and supplementary motor area are expected to critically contribute to complex fine motor function (51, 104–107). In line with these proposed models we recently detected differentially altered activation and altered functional coupling in key areas of the left praxis network to be linked to altered complex fine motor function (CR) in Parkinson’s disease (35). Likewise we detected associations of functional and structural alterations in the praxis network with gesture performance in schizophrenia patients (108–110). However, future studies are needed to address the neural correlates of impaired complex fine motor function in schizophrenia.

### Association of Motor Function With Frontal Lobe Function and Working Memory

Critical contributors to aberrant motor skills in schizophrenia are working memory and frontal lobe functioning. We, therefore, tested, whether frontal lobe function and working memory correlated with the motor domains in each group. In fact, in our study some motor domains (NSS and Parkinsonism) were associated with frontal lobe function across groups. However, there is also considerable conceptual overlap between motor skills and frontal lobe function. For instance the FAB and the NES share some items such as the fist-edge-palm test.

The pattern of correlations was different for working memory. Only in patients working memory was correlated with motor domains. This may be due to the fact that in our study working memory performance was relatively intact in relatives and healthy controls. Thus, given that motor abilities rely on frontal lobe and working memory function, working memory deficits may have impaired motor function in patients, while altered frontal lobe function compromised motor skills in all three groups. In contrast, working memory deficits were unlikely to account for motor abnormalities in relatives.

### Limitations

Some limitations of this study require discussion. The sample was predominantly female as women more frequently agreed to participation. As groups were matched for age, gender and education, the proportion of women was equal across groups. Still, former studies showed no gender effect on NSS severity in patients (111). In addition, most of our patients were medicated. Antipsychotic medication may impact motor behavior. For instance dyskinesia and Parkinsonism were typically attributed to medication (i.e., tardive dyskinesia), even though evidence suggests that these motor abnormalities may also occur spontaneously in drug naïve patients (1, 5, 10, 112–114). We used the NES to assess NSS, but whether our results are comparable to other instruments remains unclear due to inconsistent terminology across instruments (59, 115) [e.g., overlap of motor coordination (116) and sequencing of motor acts in the NES]. Finally, although we assessed a comprehensive battery of various motor domains, other important domains have not been studied. For example, we did not test postural sway (117), which is an interesting measure of cerebellar function.

### Conclusion

Modern theories for the development of psychosis suggest that environmental stressors, such as early childhood adversities, together with neurodevelopmental abnormalities may lead to psychosis. One particular marker of neurodevelopmental abnormalities is aberrant motor function. We were able to confirm generalized aberrant motor function in schizophrenia patients and aberrant motor function in some but not all tested motor domains in first-degree relatives of schizophrenia patients. The pattern of alterations in relatives suggests distinct mechanisms in each of the motor domains typically affected in psychosis. Particularly, relatives had increased NSS severity, which may reflect abnormal motor skill development. Thus, NSS could become a specific marker of the genetic risk for psychosis. Importantly such objective markers may not only inform on the etiology of psychosis but may be relevant for screening and staging of psychosis. Finally, markers of aberrant motor function may guide individualized treatment regimen in the future. Yet, future studies with sensitive technical instruments to measure and quantify different motor abnormalities are warranted to confirm our results in longitudinal studies.

## Ethics Statement

This study was carried out in accordance with the recommendations of the local Ethics Committee (Kantonale Ethikkommission: KEK Bern) with written informed consent from all subjects. All subjects gave written informed consent in accordance with the Declaration of Helsinki. The protocol was approved by the Kantonale Ethikkommission: KEK Bern.

## Author Contributions

LS contributed data acquisition, analysis and interpretation, drafting and critical revision of the manuscript. KS and SW contributed to study concept and design, study supervision, data acquisition, analysis and interpretation, drafting, and critical revision of the manuscript. SW wrote the protocol and acquired funding for the study. PV contributed to data analysis and interpretation, drafting, and critical revision of the manuscript.

## Conflict of Interest Statement

The authors declare that the research was conducted in the absence of any commercial or financial relationships that could be construed as a potential conflict of interest. The reviewer CD and handling Editor declared their shared affiliation.

## References

[1] Walther S Strik W Motor symptoms and schizophrenia. Neuropsychobiology (2012) 66(2):77–92. 10.1159/000339456 22814247

[2] Morrens M Docx L Walther S Beyond boundaries: in search of an integrative view on motor symptoms in schizophrenia. Front Psychiatry (2014) 5:145 10.3389/fpsyt.2014.00145 25352812 PMC4196470

[3] Walther S Stegmayer K Horn H Rampa L Razavi N Muller TJ The longitudinal course of gross motor activity in schizophrenia – within and between episodes. Front Psychiatry (2015) 6:10 10.3389/Fpsyt.2015.00010 25698981 PMC4318415

[4] Walther S Stegmayer K Horn H Razavi N Muller TJ Strik W. Physical activity in schizophrenia is higher in the first episode than in subsequent ones. Front Psychiatry (2015) 5:191. 10.3389/Fpsyt.2014.00191 25601842 PMC4283447

[5] Peralta V Campos MS De Jalon EG Cuesta MJ Motor behavior abnormalities in drug-naive patients with schizophrenia spectrum disorders. Mov Disord (2010) 25(8):1068–76. 10.1002/mds.23050 20222137

[6] Peralta V Cuesta MJ. Motor features in psychotic disorders. I. Factor structure and clinical correlates. Schizophr Res (2001) 47(2–3):107–16. 10.1016/S0920-9964(00)00035-9 11278127

[7] Ungvari GS Goggins W Leung SK Gerevich J. Schizophrenia with prominent catatonic features (‘catatonic schizophrenia’). II. Factor analysis of the catatonic syndrome. Prog Neuropsychopharmacol Biol Psychiatry (2007) 31(2):462–8. 10.1016/j.pnpbp.2006.11.012 17188791

[8] Chan RC Xu T Heinrichs RW Yu Y Wang Y. Neurological soft signs in schizophrenia: a meta-analysis. Schizophr Bull (2010) 36(6):1089–104. 10.1093/schbul/sbp011 19377058 PMC2963054

[9] Whitty PF Owoeye O Waddington JL. Neurological signs and involuntary movements in schizophrenia: intrinsic to and informative on systems pathobiology. Schizophr Bull (2009) 35(2):415–24. 10.1093/schbul/sbn126 18791074 PMC2659305

[10] Pappa S Dazzan P Spontaneous movement disorders in antipsychotic-naive patients with first-episode psychoses: a systematic review. Psychol Med (2008) 39(07):1065 10.1017/s0033291708004716 19000340

[11] Stuivenga M Morrens M. Prevalence of the catatonic syndrome in an acute inpatient sample. Front Psychiatry (2014) 5:174. 10.3389/fpsyt.2014.00174 25520674 PMC4253531

[12] Ungvari GS Caroff SN Gerevich J. The catatonia conundrum: evidence of psychomotor phenomena as a symptom dimension in psychotic disorders. Schizophr Bull (2010) 36(2):231–8. 10.1093/schbul/sbp105 19776208 PMC2833122

[13] Teremetz M Carment L Brenugat-Herne L Croca M Bleton JP Krebs MO Manual dexterity in schizophrenia – a neglected clinical marker? Front Psychiatry (2017) 8:120. 10.3389/fpsyt.2017.00120 28740470 PMC5502278

[14] Walther S Vanbellingen T Muri R Strik W Bohlhalter S. Impaired pantomime in schizophrenia: association with frontal lobe function. Cortex (2013) 49(2):520–7. 10.1016/j.cortex.2011.12.008 22264446

[15] Midorikawa A Hashimoto R Noguchi H Saitoh O Kunugi H Nakamura K. Impairment of motor dexterity in schizophrenia assessed by a novel finger movement test. Psychiatry Res (2008) 159(3):281–9. 10.1016/j.psychres.2007.04.004 18448171

[16] Morrens M Hulstijn W Sabbe B Psychomotor slowing in schizophrenia. Schizophr Bull (2007) 33(4):1038–53. 10.1093/schbul/sbl051 17093141 PMC2632327

[17] Heinrichs DW Buchanan RW Significance and meaning of neurological signs in schizophrenia. Am J Psychiatry (1988) 145(1):11–8. 10.1176/ajp.145.1.11 3276226

[18] Buchanan RW Heinrichs DW The neurological evaluation scale (NES): a structured instrument for the assessment of neurological signs in schizophrenia. Psychiatry Res (1989) 27(3):335–50. 10.1016/0165-1781(89)90148-0 2710870

[19] Chan RCK Cui HR Chu MY Zhang TH Wang Y Wang Y Neurological soft signs precede the onset of schizophrenia: a study of individuals with schizotypy, ultra-high-risk individuals, and first-onset schizophrenia. Eur Arch Psychiatry Clin Neurosci (2018) 268(1):49–56. 10.1007/s00406-017-0828-4 28761988

[20] Callaway D Perkins D Woods S Liu L Addington J Movement abnormalities predict transitioning to psychosis in individuals at clinical high risk for psychosis. Schizophr Bull (2015) 41:S13 10.1016/j.schres.2014.09.031 PMC4253541 25311779

[21] Neelam K Garg D Marshall M. A systematic review and meta-analysis of neurological soft signs in relatives of people with schizophrenia. BMC Psychiatry (2011) 11:139. 10.1186/1471-244x-11-139 21859445 PMC3173301

[22] Kindler J Schultze-Lutter F Michel C Martz-Irngartinger A Linder C Schmidt SJ Abnormal involuntary movements are linked to psychosis-risk in children and adolescents: results of a population-based study. Schizophr Res (2016) 174(1–3):58–64. 10.1016/j.schres.2016.04.032 27160790

[23] Mittal VA Neumann C Saczawa M Walker EF. Longitudinal progression of movement abnormalities in relation to psychotic symptoms in adolescents at high risk of schizophrenia. Arch Gen Psychiatry (2008) 65(2):165–71. 10.1001/archgenpsychiatry.2007.23 18250254

[24] Koning JP Tenback DE van Os J Aleman A Kahn RS van Harten PN. Dyskinesia and parkinsonism in antipsychotic-naive patients with schizophrenia, first-degree relatives and healthy controls: a meta-analysis. Schizophr Bull (2010) 36(4):723–31. 10.1093/schbul/sbn146 18990712 PMC2894597

[25] Tarbox SI Pogue-Geile MF. Spontaneous dyskinesia and familial liability to schizophrenia. Schizophr Res (2006) 81(2–3):125–37. 10.1016/j.schres.2005.09.013 16307868

[26] Molina JL Gonzalez Aleman G Florenzano N Padilla E Calvo M Guerrero G Prediction of neurocognitive deficits by parkinsonian motor impairment in schizophrenia: a study in neuroleptic-naive subjects, unaffected first-degree relatives and healthy controls from an indigenous population. Schizophr Bull (2016) 42(6):1486–95. 10.1093/schbul/sbw023 26994395 PMC5049519

[27] McCreadie RG Thara R Srinivasan TN Padmavathi R. Spontaneous dyskinesia in first-degree relatives of chronically ill, never-treated people with schizophrenia. Br J Psychiatry (2003) 183:45–9. 10.1192/bjp.183.1.45 12835243

[28] Makofske B Manual dexterity. In: Kreutzer J DeLuca J Caplan B, editors. Encyclopedia of Clinical Neuropsychology. New York: Springer (2011). p. 1522–3.

[29] Bervoets C Docx L Sabbe B Vermeylen S Van Den Bossche MJ Morsel A The nature of the relationship of psychomotor slowing with negative symptomatology in schizophrenia. Cogn Neuropsychiatry (2014) 19(1):36–46. 10.1080/13546805.2013.779578 23725330

[30] Docx L Morrens M Bervoets C Hulstijn W Fransen E De Hert M Parsing the components of the psychomotor syndrome in schizophrenia. Acta Psychiatr Scand (2012) 126(4):256–65. 10.1111/j.1600-0447.2012.01846.x 22360494

[31] Docx L Sabbe B Longitudinal Evaluation of the Psychomotor Syndrome in Schizophrenia. (2014). 10.1176/appi.neuropsych.13020027 26037858

[32] Pohar SL Allyson Jones C. The burden of Parkinson disease (PD) and concomitant comorbidities. Arch Gerontol Geriatr (2009) 49(2):317–21. 10.1016/j.archger.2008.11.006 19135266

[33] Foki T Vanbellingen T Lungu C Pirker W Bohlhalter S Nyffeler T Limb-kinetic apraxia affects activities of daily living in Parkinson’s disease: a multi-center study. Eur J Neurol (2016) 23(8):1301–7. 10.1111/ene.13021 27132653 PMC5565263

[34] Gebhardt A Vanbellingen T Baronti F Kersten B Bohlhalter S. Poor dopaminergic response of impaired dexterity in Parkinson’s disease: bradykinesia or limb kinetic apraxia? Mov Disord (2008) 23(12):1701–6. 10.1002/Mds.22199 18649388

[35] Kubel S Stegmayer K Vanbellingen T Pastore-Wapp M Bertschi M Burgunder JM Altered praxis network underlying limb kinetic apraxia in Parkinson’s disease – an fMRI study. Neuroimage Clin (2017) 16:88–97. 10.1016/j.nicl.2017.07.007 28765808 PMC5527158

[36] Flyckt L Sydow O Bjerkenstedt L Edman G Rydin E Wiesel FA. Neurological signs and psychomotor performance in patients with schizophrenia, their relatives and healthy controls. Psychiatry Res (1999) 86(2):113–29. 10.1016/S0165-1781(99)00027-X 10397414

[37] Gschwandtner U Pfluger M Aston J Borgwardt S Drewe M Stieglitz RD Fine motor function and neuropsychological deficits in individuals at risk for schizophrenia. Eur Arch Psychiatry Clin Neurosci (2006) 256(4):201–6. 10.1007/s00406-005-0626-2 16283597

[38] Oldfield RC The assessment and analysis of handedness: the Edinburgh inventory. Neuropsychologia (1971) 9(1):97–113. 10.1016/0028-3932(71)90067-4 5146491

[39] Sheehan DV Lecrubier Y Sheehan KH Amorim P Janavs J Weiller E The Mini-International Neuropsychiatric Interview (M.I.N.I.): the development and validation of a structured diagnostic psychiatric interview for DSM-IV and ICD-10. J Clin Psychiatry (1998) 59(Suppl 20):22–33; quiz 4–57. 9881538

[40] Dubois B Slachevsky A Litvan I Pillon B. The FAB: a frontal assessment battery at bedside. Neurology (2000) 55(11):1621–6. 10.1212/WNL.55.11.1621 11113214

[41] Wechsler D Wechsler Memory Scale (WMS-III). San Antonio, TX: Psychological Corporation (1997).

[42] Ritter N Kilinc E Navruz B Bae Y Test Review: L. Brown, R. J. Sherbenou, & S. K. Johnsen Test of Nonverbal Intelligence-4 (TONI-4). Austin, TX: PRO-ED, 2010. J Psychoeduc Assess (2011) 29(5):484–8. 10.1177/0734282911400400

[43] Guy W ECDEU Assessment Manual for Psychopharmacology, Revised ed. Washington, DC: US Department of Health, Education, and Welfare (1976). A nice practical discussion can be found in Munetz MR, Benjamin S. How to examine patients using the Abnormal Involuntary Movement Scale. *Hospital and Community Psychiatry Nov 1988, 39 (11):1172-1177* (1976). 10.1176/ps.39.11.1172 2906320

[44] Fahn S Elton RL Unified Parkinson’s disease rating scale. In: Fahn S Marsden CD Calne D Goldstein M, editors. Recent Developments in Parkinson′s Disease. Florham Park, NJ: Macmillan Healthcare Information (1987) p. 153–63.

[45] Vanbellingen T Kersten B Bellion M Temperli P Baronti F Muri R Impaired finger dexterity in Parkinson’s disease is associated with praxis function. Brain Cogn (2011) 77(1):48–52. 10.1016/j.bandc.2011.06.003 21775040

[46] Barkemeyer CA Santa Maria MP Browndyke JN Callon EB Dunn AM The coin rotation task: a convenient and sensitive measure of fine motor control. Arch Clin Neuropsychol (1998) 13(1):18 10.1016/S0887-6177(98)90379-1

[47] van Harten PN Walther S Kent JS Sponheim SR Mittal VA. The clinical and prognostic value of motor abnormalities in psychosis, and the importance of instrumental assessment. Neurosci Biobehav Rev (2017) 80:476–87. 10.1016/j.neubiorev.2017.06.007 28711662

[48] Walther S. Psychomotor symptoms of schizophrenia map on the cerebral motor circuit. Psychiatry Res (2015) 233(3):293–8. 10.1016/j.pscychresns.2015.06.010 26319293

[49] Walther S Mittal VA. Motor system pathology in psychosis. Curr Psychiatry Rep (2017) 19(12):97. 10.1007/s11920-017-0856-9 29086118

[50] Gottesman II Gould TD. The endophenotype concept in psychiatry: etymology and strategic intentions. Am J Psychiatry (2003) 160(4):636–45. 10.1176/appi.ajp.160.4.636 12668349

[51] Heilman KM Apraxia. Continuum (Minneap Minn) (2010) 16:86–98. 10.1212/01.CON.0000368262.53662.08 22810515

[52] Dutschke LL Stegmayer K Ramseyer F Bohlhalter S Vanbellingen T Strik W Gesture impairments in schizophrenia are linked to increased movement and prolonged motor planning and execution. Schizophr Res (2017) 19(12):97 10.1016/j.schres.2017.07.012 28709771

[53] Stegmayer K Moor J Vanbellingen T Bohlhalter S Muri RM Strik W Gesture performance in first- and multiple-episode patients with schizophrenia spectrum disorders. Neuropsychobiology (2016) 73(4):201–8. 10.1159/000446116 27229523

[54] Walther S Eisenhardt S Bohlhalter S Vanbellingen T Muri R Strik W Gesture performance in schizophrenia predicts functional outcome after 6 months. Schizophr Bull (2016) 42(6):1326–33. 10.1093/schbul/sbw124 27566843 PMC5049539

[55] Walther S Vanbellingen T Muri R Strik W Bohlhalter S. Impaired gesture performance in schizophrenia: particular vulnerability of meaningless pantomimes. Neuropsychologia (2013) 51(13):2674–8. 10.1016/j.neuropsychologia.2013.08.017 24001392

[56] Yazici AH Demir B Yazici KM Gogus A. Neurological soft signs in schizophrenic patients and their nonpsychotic siblings. Schizophr Res (2002) 58(2–3):241–6. 10.1016/S0920-9964(01)00338-3 12409164

[57] Chen YL Chen YH Mak FL. Soft neurological signs in schizophrenic patients and their nonpsychotic siblings. J Nerv Ment Dis (2000) 188(2):84–9. 10.1097/00005053-200002000-00004 10695836

[58] Rossi A De Cataldo S Di Michele V Manna V Ceccoli S Stratta P Neurological soft signs in schizophrenia. Br J Psychiatry (1990) 157:735–9. 10.1192/bjp.157.5.735 2132562

[59] Ismail B Cantor-Graae E McNeil TF. Neurological abnormalities in schizophrenic patients and their siblings. Am J Psychiatry (1998) 155(1):84–9. 10.1176/ajp.155.1.84 9433343

[60] Kinney DK Woods BT Yurgeluntodd D Neurologic abnormalities in schizophrenic-patients and their families 0.2. Neurologic and Psychiatric Findings in Relatives. Arch Gen Psychiatry (1986) 43(7):665–8. 10.1001/archpsyc.1986.01800070051007 3718169

[61] Compton MT Bollini AM McKenzie Mack L Kryda AD Rutland J Weiss PS Neurological soft signs and minor physical anomalies in patients with schizophrenia and related disorders, their first-degree biological relatives, and non-psychiatric controls. Schizophr Res (2007) 94(1–3):64–73. 10.1016/j.schres.2007.04.002 17512173

[62] Appels MC Sitskoorn MM de Boo M Klumpers UM Kemps A Elderson A Neurological signs in parents of schizophrenic patients. Neuroreport (2002) 13(5):575–9. 10.1097/00001756-200204160-00008 11973449

[63] Koning JP Tenback DE Kahn RS Vollema MG Cahn W van Harten PN. Movement disorders are associated with schizotypy in unaffected siblings of patients with non-affective psychosis. Psychol Med (2011) 41(10):2141–7. 10.1017/S0033291711000389 21426602

[64] Mittal VA Daley M Shiode MF Bearden CE O’Neill J Cannon TD. Striatal volumes and dyskinetic movements in youth at high-risk for psychosis. Schizophr Res (2010) 123(1):68–70. 10.1016/j.schres.2010.08.002 20732793 PMC2939316

[65] Dean DJ Mittal VA Spontaneous parkinsonisms and striatal impairment in neuroleptic free youth at ultrahigh risk for psychosis. NPJ Schizophr (2015) 1 10.1038/npjschz.2014.6 PMC4657751 26613098

[66] Hirjak D Thomann PA Kubera KM Wolf ND Sambataro F Wolf RC. Motor dysfunction within the schizophrenia-spectrum: a dimensional step towards an underappreciated domain. Schizophr Res (2015) 169(1–3):217–33. 10.1016/j.schres.2015.10.022 26547881

[67] Bernard JA Russell CE Newberry RE Goen JR Mittal VA. Patients with schizophrenia show aberrant patterns of basal ganglia activation: evidence from ALE meta-analysis. Neuroimage Clin (2017) 14:450–63. 10.1016/j.nicl.2017.01.034 28275545 PMC5328905

[68] Walther S Schappi L Federspiel A Bohlhalter S Wiest R Strik W Resting-state hyperperfusion of the supplementary motor area in catatonia. Schizophr Bull (2017) 43(5):972–81. 10.1093/schbul/sbw140 27729486 PMC5581902

[69] Woodward ND Karbasforoushan H Heckers S Thalamocortical dysconnectivity in schizophrenia. Am J Psychiatry (2012) 169(10):1092–9. 10.1176/appi.ajp.2012.12010056 23032387 PMC3810300

[70] Anticevic A Cole MW Repovs G Murray JD Brumbaugh MS Winkler AM Characterizing thalamo-cortical disturbances in schizophrenia and bipolar illness. Cereb Cortex (2014) 24(12):3116–30. 10.1093/cercor/bht165 23825317 PMC4224238

[71] Kaufmann T Skatun KC Alnaes D Doan NT Duff EP Tonnesen S Disintegration of sensorimotor brain networks in schizophrenia. Schizophr Bull (2015) 41(6):1326–35. 10.1093/schbul/sbv060 25943122 PMC4601711

[72] Walther S Stegmayer K Federspiel A Bohlhalter S Wiest R Viher PV. Aberrant hyperconnectivity in the motor system at rest is linked to motor abnormalities in schizophrenia spectrum disorders. Schizophr Bull (2017) 43(5):982–92. 10.1093/schbul/sbx091 28911049 PMC5581901

[73] Viher PV Stegmayer K Giezendanner S Federspiel A Bohlhalter S Vanbellingen T Cerebral white matter structure is associated with DSM-5 schizophrenia symptom dimensions. Neuroimage Clin (2016) 12:93–9. 10.1016/j.nicl.2016.06.013 27408794 PMC4925890

[74] Stegmayer K Horn H Federspiel A Razavi N Bracht T Laimbock K Supplementary motor area (SMA) volume is associated with psychotic aberrant motor behaviour of patients with schizophrenia. Psychiatry Res (2014) 223(1):49–51. 10.1016/j.pscychresns.2014.05.002 24853647

[75] Bracht T Schnell S Federspiel A Razavi N Horn H Strik W Altered cortico-basal ganglia motor pathways reflect reduced volitional motor activity in schizophrenia. Schizophr Res (2013) 143(2–3):269–76. 10.1016/j.schres.2012.12.004 23276479

[76] Walther S Federspiel A Horn H Razavi N Wiest R Dierks T Alterations of white matter integrity related to motor activity in schizophrenia. Neurobiol Dis (2011) 42(3):276–83. 10.1016/j.nbd.2011.01.017 21296665

[77] Walther S Federspiel A Horn H Razavi N Wiest R Dierks T Resting state cerebral blood flow and objective motor activity reveal basal ganglia dysfunction in schizophrenia. Psychiatry Res (2011) 192(2):117–24. 10.1016/j.pscychresns.2010.12.002 21511443

[78] Martinelli C Rigoli F Shergill SS. Aberrant force processing in schizophrenia. Schizophr Bull (2017) 43(2):417–24. 10.1093/schbul/sbw092 27384054 PMC5605270

[79] Hirjak D Kubera KM Thomann PA Wolf RC. Motor dysfunction as an intermediate phenotype across schizophrenia and other psychotic disorders: progress and perspectives. Schizophr Res (2017) pii:S0920-9964(17)30616-3. 10.1016/j.schres.2017.10.007 29074330

[80] Thomann PA Wustenberg T Santos VD Bachmann S Essig M Schroder J. Neurological soft signs and brain morphology in first-episode schizophrenia. Psychol Med (2009) 39(3):371–9. 10.1017/S0033291708003656 18578894

[81] Mouchet-Mages S Rodrigo S Cachia A Mouaffak F Olie JP Meder JF Correlations of cerebello-thalamo-prefrontal structure and neurological soft signs in patients with first-episode psychosis. Acta Psychiatr Scand (2011) 123(6):451–8. 10.1111/j.1600-0447.2010.01667.x 21219267

[82] Gay O Plaze M Oppenheim C Mouchet-Mages S Gaillard R Olie JP Cortex morphology in first-episode psychosis patients with neurological soft signs. Schizophr Bull (2013) 39(4):820–9. 10.1093/schbul/sbs083 22892556 PMC3686449

[83] Hirjak D Wolf RC Stieltjes B Hauser T Seidl U Schroder J Cortical signature of neurological soft signs in recent onset schizophrenia. Brain Topogr (2014) 27(2):296–306. 10.1007/s10548-013-0292-z 23660871

[84] Dazzan P Morgan KD Orr KG Hutchinson G Chitnis X Suckling J The structural brain correlates of neurological soft signs in AESOP first-episode psychoses study. Brain (2004) 127(Pt 1):143–53. 10.1093/brain/awh015 14570821

[85] Hirjak D Wolf RC Stieltjes B Seidl U Schroder J Thomann PA. Neurological soft signs and subcortical brain morphology in recent onset schizophrenia. J Psychiatr Res (2012) 46(4):533–9. 10.1016/j.jpsychires.2012.01.015 22316638

[86] Hirjak D Wolf RC Kubera KM Stieltjes B Maier-Hein KH Thomann PA. Neurological soft signs in recent-onset schizophrenia: focus on the cerebellum. Prog Neuropsychopharmacol Biol Psychiatry (2015) 60:18–25. 10.1016/j.pnpbp.2015.01.011 25640318

[87] Thomann PA Roebel M Dos Santos V Bachmann S Essig M Schroder J. Cerebellar substructures and neurological soft signs in first-episode schizophrenia. Psychiatry Res (2009) 173(2):83–7. 10.1016/j.pscychresns.2008.07.006 19540731

[88] Venkatasubramanian G Jayakumar PN Gangadhar BN Keshavan MS Automated MRI parcellation study of regional volume and thickness of prefrontal cortex (PFC) in antipsychotic-naive schizophrenia. Acta Psychiatr Scand (2008) 117(6):420–31. 10.1111/j.1600-0447.2008.01198.x 18479318

[89] Kong L Bachmann S Thomann PA Essig M Schroder J. Neurological soft signs and gray matter changes: a longitudinal analysis in first-episode schizophrenia. Schizophr Res (2012) 134(1):27–32. 10.1016/j.schres.2011.09.015 22018942

[90] Mittal VA Dean DJ Bernard JA Orr JM Pelletier-Baldelli A Carol EE Neurological soft signs predict abnormal cerebellar-thalamic tract development and negative symptoms in adolescents at high risk for psychosis: a longitudinal perspective. Schizophr Bull (2014) 40(6):1204–15. 10.1093/schbul/sbt199 24375457 PMC4193696

[91] Zhao Q Li Z Huang J Yan C Dazzan P Pantelis C Neurological soft signs are not “soft” in brain structure and functional networks: evidence from ALE meta-analysis. Schizophr Bull (2014) 40(3):626–41. 10.1093/schbul/sbt063 23671197 PMC3984512

[92] Chan RC Huang J Zhao Q Wang Y Lai YY Hong N Prefrontal cortex connectivity dysfunction in performing the Fist-Edge-Palm task in patients with first-episode schizophrenia and non-psychotic first-degree relatives. Neuroimage Clin (2015) 9:411–7. 10.1016/j.nicl.2015.09.008 26594623 PMC4596919

[93] Sarro S Pomarol-Clotet E Canales-Rodriguez EJ Salvador R Gomar JJ Ortiz-Gil J Structural brain changes associated with tardive dyskinesia in schizophrenia. Br J Psychiatry (2013) 203(1):51–7. 10.1192/bjp.bp.112.114538 23222039

[94] McCreadie RG Thara R Padmavati R Srinivasan TN Jaipurkar SD. Structural brain differences between never-treated patients with schizophrenia, with and without dyskinesia, and normal control subjects: a magnetic resonance imaging study. Arch Gen Psychiatry (2002) 59(4):332–6. 10.1001/archpsyc.59.4.332 11926933

[95] Mittal VA Orr JM Turner JA Pelletier AL Dean DJ Lunsford-Avery J Striatal abnormalities and spontaneous dyskinesias in non-clinical psychosis. Schizophr Res (2013) 151(1–3):141–7. 10.1016/j.schres.2013.10.003 24156901 PMC3855894

[96] Bai YM Chou KH Lin CP Chen IY Li CT Yang KC White matter abnormalities in schizophrenia patients with tardive dyskinesia: a diffusion tensor image study. Schizophr Res (2009) 109(1–3):167–81. 10.1016/j.schres.2009.02.003 19261444

[97] Shihabuddin L Buchsbaum MS Hazlett EA Haznedar MM Harvey PD Newman A Dorsal striatal size, shape, and metabolic rate in never-medicated and previously medicated schizophrenics performing a verbal learning task. Arch Gen Psychiatry (1998) 55(3):235–43. 10.1001/archpsyc.55.3.235 9510217

[98] Heinz A Knable MB Coppola R Gorey JG Jones DW Lee KS Psychomotor slowing, negative symptoms and dopamine receptor availability – an IBZM SPECT study in neuroleptic-treated and drug-free schizophrenic patients. Schizophr Res (1998) 31(1):19–26. 10.1016/S0920-9964(98)00003-6 9633833

[99] Mamah D Harms MP Wang L Barch D Thompson P Kim J Basal ganglia shape abnormalities in the unaffected siblings of schizophrenia patients. Biol Psychiatry (2008) 64(2):111–20. 10.1016/j.biopsych.2008.01.004 18295189 PMC2486271

[100] Cuesta MJ Lecumberri P Cabada T Moreno-Izco L Ribeiro M Lopez-Ilundain JM Basal ganglia and ventricle volume in first-episode psychosis. A family and clinical study. Psychiatry Res (2017) 269:90–6. 10.1016/j.pscychresns.2017.09.010 28963912

[101] Goldman AL Pezawas L Mattay VS Fischl B Verchinski BA Zoltick B Heritability of brain morphology related to schizophrenia: a large-scale automated magnetic resonance imaging segmentation study. Biol Psychiatry (2008) 63(5):475–83. 10.1016/j.biopsych.2007.06.006 17727823

[102] Geschwind N The apraxias: neural mechanisms of disorders of learned movement. Am Sci (1975) 63(2):188–95. 1115438

[103] Niessen E Fink GR Weiss PH. Apraxia, pantomime and the parietal cortex. Neuroimage Clin (2014) 5:42–52. 10.1016/j.nicl.2014.05.017 24967158 PMC4066186

[104] Kolb B Milner B Performance of complex arm and facial movements after focal brain lesions. Neuropsychologia (1981) 19(4):491–503. 10.1016/0028-3932(81)90016-6 7279182

[105] Haaland KY Harrington DL Knight RT. Neural representations of skilled movement. Brain (2000) 123(Pt 11):2306–13. 10.1093/brain/123.11.2306 11050030

[106] Rothi LJ Heilman KM Watson RT. Pantomime comprehension and ideomotor apraxia. J Neurol Neurosurg Psychiatry (1985) 48(3):207–10. 10.1136/jnnp.48.3.207 2580058 PMC1028251

[107] Watson RT Fleet WS Gonzalez-Rothi L Heilman KM. Apraxia and the supplementary motor area. Arch Neurol (1986) 43(8):787–92. 10.1001/archneur.1986.00520080035016 3729758

[108] Stegmayer K Bohlhalter S Vanbellingen T Federspiel A Wiest R Muri RM Limbic interference during social action planning in schizophrenia. Schizophr Bull (2018) 44(2):359–68. 10.1093/schbul/sbx059 28575506 PMC5814975

[109] Stegmayer K Bohlhalter S Vanbellingen T Federspiel A Moor J Wiest R Structural brain correlates of defective gesture performance in schizophrenia. Cortex (2016) 78:125–37. 10.1016/j.cortex.2016.02.014 27038858

[110] Viher PV Stegmayer K Kubicki M Karmacharya S Lyall AE Federspiel A The cortical signature of impaired gesturing: findings from schizophrenia. Neuroimage Clin (2018) 17:213–21. 10.1016/j.nicl.2017.10.017 29159038 PMC5683189

[111] Bombin I Arango C Buchanan RW. Significance and meaning of neurological signs in schizophrenia: two decades later. Schizophr Bull (2005) 31(4):962–77. 10.1093/schbul/sbi028 15958818

[112] Peralta V Cuesta MJ. Neuromotor abnormalities in neuroleptic-naive psychotic patients: antecedents, clinical correlates, and prediction of treatment response. Compr Psychiatry (2011) 52(2):139–45. 10.1016/j.comppsych.2010.05.009 21295219

[113] Peralta V Cuesta MJ The effect of antipsychotic medication on neuromotor abnormalities in neuroleptic-naive nonaffective psychotic patients: a naturalistic study with haloperidol, risperidone, or olanzapine. Prim Care Companion J Clin Psychiatry (2010) 12(2). 10.4088/PCC.09m00799gry PMC2911000 20694120

[114] Stegmayer K Walther S van Harten P Tardive dyskinesia associated with atypical antipsychotics: prevalence, mechanisms and management strategies. CNS Drugs (2018) 32(2):135–47. 10.1007/s40263-018-0494-8 29427000

[115] Mechri A Slama H Bourdel MC Chebel S Mandhouj O Krebs MO [Neurological soft signs in schizophrenic patients and their nonaffected siblings]. Encephale (2008) 34(5):483–9. 10.1016/j.encep.2007.08.009 19068337

[116] Krebs MO Gut-Fayand A Bourdel MC Dischamp J Olie JP. Validation and factorial structure of a standardized neurological examination assessing neurological soft signs in schizophrenia. Schizophr Res (2000) 45(3):245–60. 10.1016/S0920-9964(99)00206-6 11042442

[117] Dean DJ Kent JS Bernard JA Orr JM Gupta T Pelletier-Baldelli A Increased postural sway predicts negative symptom progression in youth at ultrahigh risk for psychosis. Schizophr Res (2015) 162(1–3):86–9. 10.1016/j.schres.2014.12.039 25601361 PMC4339540

